# Gender Differences Relating to Metabolic Syndrome and Proinflammation in Finnish Subjects with Elevated Blood Pressure

**DOI:** 10.1155/2009/959281

**Published:** 2009-08-20

**Authors:** Tiina Ahonen, Juha Saltevo, Markku Laakso, Hannu Kautiainen, Esko Kumpusalo, Mauno Vanhala

**Affiliations:** ^1^Palokka Health Center, 40270 Jyväskylä, Finland; ^2^Department of Medicine, Central Finland Central Hospital, 40600 Jyväskylä, Finland; ^3^Department of Medicine, Kuopio University Hospital, University of Kuopio, 70210 Kuopio, Finland; ^4^ORTON, Rehabilitation Unit, 00280 Helsinki, Finland; ^5^Unit of General Practice, Central Finland Central Hospital, 40600 Jyväskylä, Finland; ^6^Department of Public Health and Clinical Nutrition, University of Kuopio, 70210 Kuopio, Finland; ^7^Unit of Family Practice, Kuopio University Hospital, 70210 Kuopio, Finland

## Abstract

Fasting insulin, adiponectin, high-sensitivity C-reactive protein (hs-CRP), and interleukin-1 receptor antagonist (IL-1Ra) were determined in 278 men and 273 women with blood pressure ≥130 and/or ≥85 mmHg and/or with antihypertensive medication. Metabolic syndrome (MetS) with the National Cholesterol Education Program (NCEP) criteria was observed in 35% of men and 34% of women. Men with MetS had lower hs-CRP and IL-1Ra than women. The absolute gender difference in adiponectin was smaller and those in IL-1Ra and hs-CRP were greater in subjects with MetS compared to those without. After adjustment with body mass index the association between insulin and the odd's ratio (OR) for MetS remained significant in both genders, in females also the association between the OR for MetS and adiponectin. There are gender differences in subjects with elevated blood pressure and MetS with respect to inflammatory markers and the relationship between adiponectin levels and MetS.

## 1. Introduction

Elevated blood pressure (BP) is associated with insulin resistance or hyperinsulinemia [1–4] and hypertension has been included in the definition of metabolic syndrome (MetS) [5]. MetS, that is, a cluster of cardiovascular risk factors accompanied by an increased risk of cardiovascular disease (CVD) and type 2 diabetes, is common in obese subjects with a sedentary lifestyle [6–9]. Similarly, smokers and heavy alcohol users often have MetS [10, 11].

Adipose tissue secretes a variety of inflammatory markers associated with elevated BP, insulin resistance, and MetS [12]. Adiponectin concentrations are low in subjects with essential hypertension [13], and hypoadiponectinemia has been considered to be a potential additional component of MetS [14, 15]. High levels of high-sensitivity C-reactive protein (hs-CRP) are often observed in hypertensive subjects [16] and in subjects with MetS [17]. Elevated levels of interleukin-1*β* (IL-1*β*) and interleukin-1 receptor antagonist (IL-1 Ra) have been detected in subjects with essential hypertension [18–21] and they are known to contribute to the development of insulin resistance and MetS [18, 22]. The IL-1 Ra level has been shown to be the most sensitive marker for cytokine response in the prediabetic state [23]. 

MetS has been shown to predict CVD events in hypertensive subjects independently of traditional risk factors [24]. MetS in hypertensive subjects impairs total arterial compliance, and MetS in subjects with untreated hypertension has been shown to be associated with aortic stiffness [25, 26]. 

Earlier it has been shown that there are gender differences in the levels of hs-CRP and IL-1 Ra among subjects with MetS [27]. However there are limited data on gender differences with respect to CVD and risk factors for CVD in hypertensive subjects with or without MetS. MetS seems to be a stronger predictor of CVD in women than in men [28]. Markers of inflammation and fibrosis have been shown to be related to cardiovascular damage in hypertensive subjects with MetS [29]. The effect of MetS on left ventricular function and hypertrophy is greater in women than in men [30]. Furthermore, a high level of hs-CRP is a better predictor of type 2 diabetes in women than in men [31].

To further investigate the possible gender differences relating to MetS and proinflammatory markers, adiponectin and insulin resistance, we studied this question in a population-based sample of subjects with elevated blood pressure, with or without MetS.

## 2. Materials and Methods

The study population consisted of 1294 subjects from Pieksämäki, Eastern Finland. Out of all subjects, who were born in 1942, 1947, 1952, 1957, and 1962 and invited for a health check-up, a total of 923 (71.3%) decided to participate in the study. The study protocol was approved by the Ethics Committee of Kuopio University Hospital and the University of Kuopio. All participants gave an informed written consent.

All subjects having systolic BP ≥ 130 mmHg or diastolic BP ≥ 85 mmHg or receiving antihypertensive medication were included in the statistical analyses, while subjects with hs-CRP ≥ 10 pg/mL (*n* = 18) were excluded from the analysis because of the possibility of acute infections. The final study population consisted of 551 subjects (278 men, 273 women).

All subjects filled in a questionnaire about their medication, smoking habits, alcohol consumption, and physical activity. They were also interviewed by a trained nurse. Subjects who smoked on a daily basis were considered to be current smokers. All subjects who used alcohol, regardless of the amount, were considered to be alcohol users. Subjects exercising in their free time at least three times a week with a minimum of 30 minutes a time were considered to be physically active.

BP was measured by a nurse with a mercury sphygmomanometer in a sitting position after 15 minutes of rest. The measurement was repeated after five minutes. The mean of the two measurements was used in the statistical analyses. Waist circumference was measured from the midpoint between the lateral iliac crest and the lowest rib to an accuracy of 0.5 cm. Weight and height were measured to an accuracy of 0.1 kg and 0.5 cm, respectively. Body mass index (BMI) was calculated as weight (kg) divided by height (m) squared.

The MetS was defined by the National Cholesterol Education Program (NCEP) Expert Panel on Detection, Evaluation and Treatment of High Blood Cholesterol in Adults (ATP III) criteria. Subjects with three or more of the following components were classified as having MetS: (1) increased waist circumference (≥102 cm ≥40 in for men and ≥88 cm ≥35 in for women), (2) elevated fasting total triglycerides ( ≥1.7 mmol/l ≥150 mg/dl or treatment for dyslipidemia), (3) low fasting serum high density lipoprotein (HDL) cholesterol (<1.03 mmol/l <40 mg/dl in men or <1.29 mmol/l <50 mg/dl in women or treatment for dyslipidemia), (4) systolic BP ≥130 mmHg or diastolic BP ≥85 mmHg or the use of antihypertensive medication, and (5) fasting plasma glucose ≥5.6 mmol/l ≥100 mg/dl or the use of antihyperglycemic medication. 

Fresh serum samples were drawn after an overnight fast. Plasma was separated by centrifugation for the determination of fasting insulin, and the samples were frozen immediately. Plasma insulin was determined using the Phadeseph Insulin radioimmunoassay (RIA) 100 method (Pharmacia Diagnostics AB, Uppsala, Sweden). Plasma glucose concentration was measured by an automated colorimetric method (Peridochrom Glucose GOD-PAP, Boehringer, Germany). Serum triglycerides were measured from fresh serum samples by enzymatic colorimetric methods (CHOD-PAP, GPO-PAP, Boehringer Mannheim GmbH, Germany). Serum HDL cholesterol was measured by the same method after precipitation of low density lipoprotein cholesterol and very low density lipoprotein cholesterol with phosphotungstic acid and magnesium. Hs-CRP was measured with an Immulite analyzer and a DPC high-sensitivity CRP assay (DPL, Los Angeles, CA, USA). Serum adiponectin was determined with an enzyme immunoassay (Human Adiponectin ELISA Kit, B-Bridge International INC, Mountains View, CA, USA). Plasma concentrations of IL-1Ra were determined with high-sensitivity assay kits from R&D systems (Minneapolis, MN,USA).

## 3. Statistical Analysis

The results are expressed as mean ± standard deviation (SD). The most important outcomes are given with 95 per cent confidence intervals (95% CI). The Clopper-Pearson method was used to calculate the confidence interval for the prevalence rate. The confidence intervals for odds ratios were obtained by bias-corrected bootstrapping (5000 replications) [32]. Statistical comparisons between the groups were performed by chi-square test, *t*-test, or bootstrap-type *t*-test as appropriate. Logistic regression models were used to investigate the linear association between MetS and gender-specific tertiles of insulin, hs-CRP, adiponectin and inflammatory cytokines. We controlled for age, physical activity, smoking, and use of alcohol in the model 1 and added BMI for the model 2. The normality of variables was evaluated by Shapiro-Wilk statistics. No adjustment was made for multiple testing, but this information can be obtained by multiplying the actual *P*-value by the number of comparisons made.

## 4. Results

The study population consisted of 278 men and 273 women with elevated blood pressure. There were no statistically significant gender differences in the use of antihypertensive medication (14.4% of men, 14.7% of women, *P* = .930 between the genders). Mostly used were ace-inhibitors and B-blockers with no gender differences. In men, waist circumference, diastolic BP, triglyceride levels, and fasting glucose were significantly higher (*P* < .001) and the level of HDL cholesterol was significantly lower (*P* < .001) compared to women. The number of current smokers and regular alcohol users was significantly higher among men (*P* < .001) than women (Table 1).

MetS was observed in 35% (95% CI: 30% to 41%) of the men and 34% (95% CI: 29% to 40%) of the women (*P* = .84 between the genders). There were no statistically significant gender differences in the mean BMI regardless of whether the subjects had MetS (30.0 ± 3.8 in men versus 30.9 ± 5.1 kg/m^2^ in women, *P* = .20) or not (25.6 ± 2.7 in men versus 25.2 ± 4.0 kg/m^2^ in women, *P* = .27 between the genders). 

Table 2 shows the comparison of the levels of fasting insulin, hs-CRP, and cytokines between the genders in subjects with and without MetS. In men without MetS, adiponectin levels were lower compared to those in women without MetS [−3.85 *μ*g/mL (95% CI: −4.72 to −3.10), *P* < .001]. In subjects with MetS, the gender difference in adiponectin levels between men and women was −2.53 *μ*g/mL (95% CI: −3.44 to −1.68 *μ*g/mL, *P* < .001). In subjects without MetS, no statistically significant gender differences were observed in any other parameters measured, but in women with MetS the level of hs-CRP was 0.67 pg/mL (95% CI: 0.13 to 1.17 pg/mL, *P* = .013) and the level of IL-1Ra was 77 pg/mL (95% CI: 17 to 141 pg/mL, *P* = .014) higher than the corresponding levels seen in men. 

Table 3 presents the association of gender-specific tertiles of fasting insulin, hs-CRP, adiponectin, and proinflammatory cytokines with the presence of MetS by gender in two adjusted models. In men, the odd's ratio (OR) for MetS was 22.02 (95% CI: 9.20 to 52.71) in the highest tertile of fasting insulin compared to the lowest tertile after adjustment for age, physical activity, smoking status, and alcohol use ( Model 1). The corresponding ORs relating to adiponectin and IL-1Ra were 0.25 (95% CI: 0.13 to 0.49) and 2.61 (95% CI: 1.37 to 4.94), respectively. After further adjustment for BMI (Model 2), only the association of fasting insulin with the presence of MetS remained statistically significant. In women, the adjusted OR (Model 1) for MetS was 17.71 (95% CI: 7.54 to 41.59) in the highest tertile of insulin compared to the lowest tertile. The ORs relating to hs-CRP, adiponectin and IL-1Ra were 4.85 (95% CI: 2.47 to 9.52), 0.25 (95% CI: 0.13 to 0.50), and 6.80 (95% CI: 3.25 to 14.21), respectively. After further adjustment for BMI in women (Model 2), the associations between the presence of MetS and fasting insulin and adiponectin remained statistically significant.

## 5. Discussion

The novel finding of our study was the gender difference in the association of the levels of adiponectin and other inflammatory markers with MetS defined by the NCEP criteria in subjects with elevated blood pressure. Women with MetS had higher levels of proinflammatory markers compared to men. Furthermore, absolute differences in adiponectin, hs-CRP, and IL-1Ra levels in subjects with and without MetS were greater in women than in men. The association between adiponectin levels and the presence of MetS was statistically significant even after adjustment for BMI in women but not in men.

Hypoadiponectinemia has been shown to be associated with insulin resistance, coronary artery disease, and central obesity [12, 13]. Adiponectin is expressed more in subcutaneous fat than in visceral fat [12], which explains at least in part the gender difference in adiponectin levels in the general population. Similar results were also obtained in our study. Furthermore, in subjects with MetS the decrease in adiponectin levels was relatively greater in women than in men, and in subjects with MetS the absolute gender difference in adiponectin levels was about 1.3 *μ*g/ml lower than that observed in subjects without MetS. Decreased adiponectin synthesis has been assumed to lead to dysregulation of the control mechanisms inhibiting the production of proinflammatory cytokines [33]. Thus, the greater decrease in adiponectin levels observed in women could lead to more proinflammation compared to men. Our finding of higher absolute and relative cytokine levels in women with MetS supports this hypothesis.

In our study, a 4-fold higher probability for the presence of MetS was observed in the lowest adiponectin tertile compared to the highest adiponectin tertile in both genders. After adjustment for BMI, this association disappeared in men but remained statistically significant (3-fold) in women. The greater reduction in adiponectin levels seen in women with MetS may imply that the role of adiponectin in MetS is more important in women than in men, at least in subjects with elevated blood pressure. This finding could be explained by differences in body composition. Compared to women, men have relatively more muscle mass and less total body fat, which is also more viscerally located. In women, fat is known to accumulate in the subcutaneous compartment, especially in the gluteal-femoral region [34]. However, increasing accumulation of visceral fat has also been observed in women with MetS [35], which probably leads to a decrease in adiponectin production**.**

IL-1*β* is one of the major proinflammatory cytokines synthesized mainly by monocytes and macrophages outside the fat cell in adipose tissue [18]. Its expression is increased in visceral adipose tissue in obese subjects [36] and in patients with essential hypertension [19]. IL-1*β* stimulates the release of IL-1Ra which antagonizes IL-1*β* binding to the cell receptors [20, 36]. IL-1*β* levels are not usually elevated in plasma while anti-inflammatory IL-1Ra is constantly present in the circulation [20]. Increases in IL-1*β* are often associated with increases in hs-CRP [37], which is synthesized in the liver in response to IL-1*β*. In our study, women with MetS had higher absolute levels of hs-CRP and IL-1Ra compared to men with MetS. A statistically significant gender difference in the ratio of hs-CRP levels between subjects with and without MetS was detected in our study, indicating that the absolute increase in hs-CRP in women with MetS was greater than that in men with MetS. The gender differences in hs-CRP levels vary in different studies in the Hoorn study significant association of hs-CRP with incident diabetes mellitus was observed in men, but not in women [38]. In Japanese subjects this association was observed in both sexes [39], but solely in women in the Mexico City Diabetes Study [40] and in the MONICA/KORA study [31]. 

Furthermore, there was a larger relative decrease in the adiponectin level and a larger relative increase in IL-1Ra levels in women with MetS compared to men with MetS. All these findings are in line with previously reported disturbances in the regulation of inflammatory cytokines associated with decreased adiponectin levels [33]. 

Elevated plasma insulin is widely used as a marker of insulin resistance [41], at least in large population studies where more accurate methods such as the insulin clamp technique cannot be used. Excessive mobilization of free fatty acids takes place in the visceral adipose tissue through a higher rate of lipolysis in visceral fat compared to subcutaneous fat. High levels of free fatty acids inhibit insulin-mediated glucose uptake in skeletal muscle leading to insulin resistance [42, 43]. The increase in circulating free fatty acids also increases insulin secretion leading to hyperinsulinemia. In our study, insulin resistance measured by fasting insulin was similarly associated with MetS in both genders, showing a 22-fold risk in men and an 18-fold risk in women in the highest tertile of fasting insulin compared to the lowest tertile. This risk remained statistically significant even after adjustment for BMI emphasizing the central role of insulin resistance in MetS.

As far as we know, this is the first report in subjects with elevated blood pressure showing a gender difference in the levels of IL-1Ra and hs-CRP in subjects with MetS defined by the NCEP criteria and a gender difference in the relation between adiponectin and the presence of MetS. The strength of our study is that our sample included five entire age groups from one town. The number of subjects receiving antihypertensive medication was small, meaning that medication is not likely to bias our results. The relatively small number of subjects with MetS is a limitation of our study. 

We conclude that gender differences in inflammatory markers and the relationship between adiponectin levels and MetS exist in subjects who have elevated blood pressure and MetS. The significance of these findings necessitates further research.

## Figures and Tables

**Table 1:** Demographic, clinical, biochemical, and lifestyle factors of subjects.

Variables	Men	Women	*P*-value
	(*N* = 278)	*N* = 273	
Demographic			
* *Age, years	47 (6)	48 (6)	0.38
* *Age distribution, years	36–57	36–57	NS
* *Body mass index, kg/m^2^	27.1 (3.8)	27.1 (5.2)	.98
* *Waist circumference, cm	95 (11)	85 (13)	<.001
Clinical			
* *Blood pressure, mmHg			
* *Systolic	146 (15)	144 (14)	.10
* *Diastolic	88 (9)	85 (8)	<.001
Medication for hypertension (%)	40 (14)	40 (15)	.93
Medication for hyperlipidemia (%)	12 (4.4)	5 (2.0)	.092
Medication for diabetes (%)	6 (2.0)	7 (3.0)	.75
Biochemical			
* *Total cholesterol, mmol/l	5.9 (1.1)	5.7 (1.0)	.11
* *HDL cholesterol, mmol/l	1.3 (0.3)	1.5 (0.4)	<.001
* *Triglycerides, mmol/l	1.7 (1.4)	1.3 (0.6)	<.001
* * fP-glucose, mmol/l	6.0 (0.7)	5.7 (0.6)	<.001
Lifestyle			
* *Number of current smokers (%)	94 (34)	52 (19)	<.001
* *Number of alcohol users (%)	243 (88)	212 (78)	<.001
* *Number of physically active (%)	87 (31)	78 (27)	.48

HDL: high density lipoprotein; fP: fasting plasma. (mean SD in parentheses for continuous variables number of subjects, percentage in parentheses for dichotomous variables).

**Table 2:** Insulin, hs-CRP, and cytokines according to the presence of metabolic syndrome.

Variables	MetS not present			MetS present		
	Men (*n* = 181)	Women (*n* = 180)	*P* value^†^	Men (*n* = 97)	Women (*n* = 93)	*P* value^†^
	Mean (SD)	Mean (SD)		Mean (SD)	Mean (SD)	

fP-insulin, mU/L	9.2 (4.4)	8.4 (2.8)	.065	15.3 (7.8)	15.0 (12.4)	.85
hs-CRP, pg/mL	1.32 (1.58)	1.25 (1.37)	.65	1.68 (1.70)	2.35 (1.99)	.013
Adiponectin, *μ*g/mL	5.02 (2.50)	8.87 (4.84)	<.001	4.11 (2.36)	6.64 (3.72)	<.001
IL-1Ra, pg/mL	157 (81)	174 (147)	.17	212 (208)	289 (242)	.014

^†^Bootstrap type *t*-test. MetS: metabolic syndrome defined by the NCEP criteria. fp: fasting insulin. hs-CRP: high sensitive C-reactive protein. IL-1Ra: interleukin 1-receptor antagonist.

**Table 3:** Odds ratio (OR) for the presence of metabolic syndrome defined by the NCEP criteria according to the gender-specific tertiles of insulin, C-reactive protein, adiponectin and interleukin-1- receptor antagonist in men and women in two adjusted models.

Variables	Men				Women			
	Model 1		Model 2		Model 1		Model 2	
	OR (95% CI)	*P*-value^†^	OR (95% CI)	*P*-value^†^	OR (95% CI)	*P*-value	OR (95% CI)	*P*-value^†^
fP-insulin		<001		<.001		<.001		.013
1st tertile	1.00^‡^		1.00		1.00		1.00	
2nd tertile	4.77 (2.01 to 11.34)		3.76 (1.46 to 9.66)		4.61 (1.95 to 10.88)		3.15 (1.27 to 7.80)	
3rd tertile	22.02 (9.20 to 52.71)		5.98 (2.20 to 16.23)		17.71 (7.54 to 41.59)		6.00 (2.36 to 15.27)	
hs-CRP		.12		.26		<.001		.22
1st tertile	1.00		1.00		1.00		1.00	
2nd tertile	1.58 (0.84 to 2.97)		0.98 (0.45 to 2.11)		1.76 (0.87 to 3.55)		1.21 (0.55 to 2.65)	
3rd tertile	1.65 (0.87 to 3.10)		0.63 (0.28 to 1.42)		4.85 (2.47 to 9.52)		1.65 (0.74 to 3.66)	
Adiponectin		<.001		.44		<.001		.003
1st tertile	1.00		1.00		1.00		1.00	
2nd tertile	0.32 (0.17 to 0.61)		0.67 (0.30 to 1.47)		0.59 (0.32 to 1.09)		0.67 (0.33 to 1.37)	
3rd tertile	0.25 (0.13 to 0.49)		0.73 (0.33 to 1.61)		0.25 (0.13 to 0.50)		0.30 (0.14 to 0.67)	
IL-1Ra		.003		.77		<.001		.068
1st tertile	1.00		1.00		1.00		1.00	
2nd tertile	1.68 (0.88 to 3.22)		0.94 (0.43 to 2.06)		3.98 (1.88to 8.43)		2.45 (1.07 to 5.62)	
3rd tertile	2.61 (1.37 to 4.94)		1.12 (0.52 to 2.44)		6.80 (3.25 to 14.21)		2.22 (0.94 to 5.24)	

Model 1: adjusted for age, physical activity, smoking status, and alcohol use. Model 2: adjusted for age, physical activity, smoking status, alcohol use, and BMI. ^†^ *P*-value for linearity. ^‡^Denominator of odds ratio.
